# Virulence Genes among *Enterococcus faecalis* and *Enterococcus faecium* Isolated from Coastal Beaches and Human and Nonhuman Sources in Southern California and Puerto Rico

**DOI:** 10.1155/2016/3437214

**Published:** 2016-04-10

**Authors:** Donna M. Ferguson, Ginamary Negrón Talavera, Luis A. Ríos Hernández, Stephen B. Weisberg, Richard F. Ambrose, Jennifer A. Jay

**Affiliations:** ^1^Department of Environmental Health Sciences, University of California, Los Angeles, Room 46-081 CHS, P.O. Box 951772, Los Angeles, CA 90095-1772, USA; ^2^University of Puerto Rico at Mayaguez, Biology Building, Road 108, Km 1, Mayaguez, PR 00680, USA; ^3^Southern California Coastal Water Research Project, 3535 Harbor Boulevard, Suite 110, Costa Mesa, CA 92626, USA; ^4^Department of Civil and Environmental Engineering, University of California, Los Angeles, 5732H Boelter Hall, Los Angeles, CA 90095-1593, USA

## Abstract

Most* Enterococcus faecalis* and* E. faecium* are harmless to humans; however, strains harboring virulence genes, including* esp, gelE, cylA, asa1*, and* hyl*, have been associated with human infections.* E. faecalis* and* E. faecium* are present in beach waters worldwide, yet little is known about their virulence potential. Here, multiplex PCR was used to compare the distribution of virulence genes among* E. faecalis* and* E. faecium* isolated from beaches in Southern California and Puerto Rico to isolates from potential sources including humans, animals, birds, and plants. All five virulence genes were found in* E. faecalis* and* E. faecium* from beach water, mostly among* E. faecalis*.* gelE* was the most common among isolates from all source types. There was a lower incidence of* asa1*,* esp*,* cylA*, and* hyl* genes among isolates from beach water, sewage, septage, urban runoff, sea wrack, and eelgrass as compared to human isolates, indicating that virulent strains of* E. faecalis* and* E. faecium* may not be widely disseminated at beaches. A higher frequency of* asa1* and* esp* among* E. faecalis* from dogs and of* asa1* among birds (mostly seagull) suggests that further studies on the distribution and virulence potential of strains carrying these genes may be warranted.

## 1. Introduction

*Enterococcus faecalis* and* Enterococcus faecium* are commonly found in the intestinal tracts of humans and animals and also ubiquitous in the environment [1]. While generally considered to be harmless, certain strains of* E. faecalis* and* E. faecium* are among the leading causes of nosocomial infections including urinary tract infections, abdominal and wound infections, endocarditis, and bacteremia [2–5].* E. faecalis* and* E. faecium* isolated from patients in hospital settings have been shown to harbor a higher frequency of* gelE* (gelatinase),* asa1* (aggregation substance),* esp* (enterococcal surface protein),* cylA* (cytolysin activator), and* hyl* (glycoside-hydrolase) as compared to strains found in nonhospitalized individuals, animals, and food [6–12]. Commensal, that is, harmless,* E. faecalis* and* E. faecium* can become opportunistic pathogens by acquiring antibiotic resistant and putative virulent genes from other bacteria via horizontal gene transfer [2, 4, 13–17].

*E. faecalis* and* E. faecium* are among the most common species of enterococci found in the beach environment [18–21]. Enterococci found in the beach environment can include naturalized populations existing in soil and vegetation as well as strains from humans, sewage, animals, birds, reptiles, and insects [1]. Presumably, potentially pathogenic* E. faecalis* and* E. faecium* in human fecal waste would harbor higher numbers of virulence genes as compared to strains from animal and environmental sources.

In Puerto Rico, beaches receive storm flows containing contaminated septage and agricultural runoff potentially carrying enterococci derived from human and animal fecal waste. In Southern California, urban runoff, beach sand, and sea wrack (macroalgae on beach sand) have been identified as important sources of enterococci to beach water [22–25].

Previous studies showed that* E. faecalis* and* E. faecium* from the beach water and sand harbor antibiotic resistant genes suggesting a potential health risk for beach goers [26–30]; however, the frequency of other virulence factors was not determined. Here, we compared the frequency of putative enterococcal virulence genes (*esp*,* gelE*,* cylA*,* asa1*, and* hyl*) among* E. faecalis* and* E. faecium* from beaches in Southern California and Puerto Rico impacted by different enterococci source inputs to assess beaches as an environmental reservoir of potentially virulent enterococci.

## 2. Materials and Methods

### 2.1. Sources of* E. faecalis* and* E. faecium* Isolates

#### 2.1.1. Southern California

A total of 170* Enterococcus* (91* E. faecalis* and 79* E. faecium*) isolates were screened for putative enterococcal virulence genes (Table 1). The environmental isolates were randomly selected from a collection of strains obtained from previous studies [20, 31] including beach water, eelgrass (*Zostera marina*), wrack (mainly* Macrocystis pyrifera*), sand, creek, or storm drain runoff upstream of beaches and sewage influent (untreated waste) and effluent. Isolates from animals were obtained from bird (mostly seagull) stools on beach and dog stools. Human (nonclinical) strains of* E. faecalis* and* E. faecium* were isolated from urine and fecal samples from 18 healthy (nonhospitalized) individuals residing in Southern California. Human fecal and urine specimens obtained from healthy individuals were considered representative of strains that could be found in beach water due to human shedding or contamination from sewage and/or septage. Ten* E. faecium* isolates identified as vancomycin resistant enterococci (VRE), 5 clinical strains of* E. faecium* (non-VRE), and 10* E. faecalis* isolated from rectal swabs (3), urine (1), blood (1), abscess (1), ascites (2), vagina (1), and joint (1) were provided by Orange County Public Health Laboratory (OCPHL). Clinical isolates were included for comparison to strains with enhanced virulence potential.

#### 2.1.2. Puerto Rico

A total of 247* Enterococcus* (174* E. faecalis* and 73* E. faecium*) isolates from Puerto Rico were analyzed (Table 2). Enterococcal isolates from beach water were obtained from two beaches in Puerto Rico. Human (nonclinical) enterococcal strains were isolated from fresh fecal samples from nine healthy individuals from Mayaguez, Puerto Rico. Clinical enterococcal strains were isolated from urine specimens and identified to species level by a local hospital in Mayaguez. Six septage samples were obtained from individual houses or from septic tank trucks after emptying individual family tanks.

### 2.2. Isolation and Identification of Enterococci

#### 2.2.1. Southern California

Enterococcal isolates from all samples (except for clinical specimens) were obtained using mEI agar and identified to species level using the Vitek II (bioMérieux) plus additional biochemical tests and pigment and motility as per Ferguson et al. [32]. Clinical strains were isolated by OCPHL using TSA with 5% sheep's blood; presumptive enterococcal colonies were gram-stained and identified using MicroScan (Siemens Healthcare) and/or API Strep 20 (bioMérieux). Up to 3 isolates per sample identified as* E. faecalis* and* E. faecium* were randomly selected for virulence gene analysis. Species identification of 8 different isolates obtained using biochemical methods was also confirmed by 16S rRNA sequencing conducted at GenoSeq, University of California, Los Angeles.

#### 2.2.2. Puerto Rico

Enterococcal isolates from all samples (except for clinical specimens) were obtained using mE agar. All isolates were divided into four groups based on pigmentation and motility. The isolates were identified to the genus level based on growth in BHI with 6.5% NaCl, growth at 45°C, esculin hydrolysis, catalase, and PCR amplifying of the Tuf gene [33]. Species level identification was done by a double digestion of the PCR product of the ATP synthase *α* subunit gene in combination with a restriction fragment length polymorphism (RFLP) assay (paper in preparation). Clinical strains obtained from a local hospital were identified using MicroScan system (Siemens Health Care). Up to 12 isolates each of* E. faecalis* or* E. faecium* were randomly selected per sample for virulence gene analysis.

### 2.3. DNA Extraction

#### 2.3.1. Southern California and Puerto Rico

*E. faecalis* and* E. faecium* strains were grown in BHI broth, incubated overnight at 37°C, and harvested by centrifugation (13,000 RPM for 5 min). The cells were washed three times in TE buffer and resuspended in 200 *μ*L 1x TE (10 mM Tris-HCl; 1 mM EDTA, pH 8.0) and lysed by heating at 95°C for 10 min. The lysed cells were transferred to tubes with glass beads, subjected to bead beating for five minutes, and centrifuged as before.

### 2.4. Multiplex PCR for the Detection of Enterococcal Virulence Genes

Total DNA extracted from all isolates obtained from California and Puerto Rico was screened for enterococcal virulence genes (*gelE*,* asa1*,* esp*,* cylA*, and* hyl*) using PCR primers and multiplex method developed by Vankerckhoven et al. [34] with the following modifications: we used Promega Flexi Taq DNA polymerase instead of Hot-StarTaq DNA polymerase in the master mix; the initial activation step was done at 95°C for 2 min, followed by 35 cycles of denaturation (95°C for 30 sec), annealing (49.5°C for 30 sec), and extension (72°C for 2 min) and 1 cycle of elongation at 72°C for 10 min. Each set of primers has a characteristic product size to differentiate within the five virulence genes (*asa1* at 375 bp,* gelE* at 213 bp,* cylA* at 688 bp,* esp* at 510 bp, and* hyl* at 276 bp). PCR products obtained by the Puerto Rico laboratory were confirmed by 1.8% agarose-gel electrophoresis (90 v, 2.5 hrs), stained with ethidium bromide, and visualized by UV transillumination (VersaDoc MP 4000). In Southern California, PCR products were visualized using the FlashGel® (Lonza) system. 2 *μ*L of extracted DNA was diluted in 2 *μ*L FlashGel loading dye and inserted into 12 + 1-cassette wells. A 50 bp–1.5 kb DNA ladder (Lonza) was used as a molecular size marker. FlashGels were run at 150 V for up to 13 minutes. Each PCR run included a no-template control; the positive control strain used for* gelE*,* esp*,* asa1*, and* cylA* was* E. faecalis* MMH594 kindly donated by N. Shankar, Department of Medicinal Chemistry and Pharmaceutics, University of Oklahoma Health Sciences Center, Oklahoma City [14].

## 3. Results

A total of 170* E. faecalis* and* E. faecium* isolates from Southern California (SC) and 247 isolates from Puerto Rico (PR) from beach water and potential sources of enterococci to beaches were analyzed for enterococcal virulence genes* gelE*,* asa1*,* esp*,* cylA*, and* hyl*.

Eighty-seven (80.6%)* E. faecalis* isolates from PR beach water harbored one or more of the following genes:* gelE* (98.1%),* asa1* (44.4%),* esp* (11.1%), and* cylA* (3.3%) (Table 3). Five (26.3%)* E. faecalis* isolates from septage contained* gelE* (21.0%),* asa1* (5.3%),* cylA* (5.3%), and* hyl* (5.3%). Eighteen (91.7%)* E. faecalis* isolates from human specimens (clinical and nonclinical) contained* gelE* (17.4–100%),* asa1* (50–100%),* esp* (33.3–40%), and* cylA* (19–60%).

Eight (100%)* E. faecalis* isolates from SC beach water contained one or more of the following genes:* gelE* (100%),* asa1* (12.5%), and* cylA* (12.5%) (Table 3). Fourteen (70%)* E. faecalis* isolates from urban runoff contained* gelE* (70%),* asa1* (10%), and* esp* (1%).* gelE* was present among wrack and eelgrass; however, the other virulence genes were rare or absent in occurrence.* E. faecalis* from dogs contained* gelE* (85.7%),* asa1* (42.9%), and* esp* (28.6%); isolates from birds contained* gelE* (100%) and* asa1* (55.5%).

The frequency of enterococcal virulence genes differed between* E. faecalis* and* E. faecium*. Virulence genes were absent among the majority of* E. faecium* beach water isolates from PR and SC (62.5% and 100%), respectively (Table 4). Interestingly, enterococcal virulence genes were also rare among* E. faecium* from sewage from SC and septage from PR.* esp* was the most common virulence gene found among* E. faecium* from humans (12.5% to 83.3%). Ten clinical isolates of* E. faecium* from SC that were identified as vancomycin resistant strains by OCPHL were positive for the* esp* (80%) and* hyl* (10%) genes. In SC, none of the 5 virulence genes were detected among* E. faecium* isolates obtained from dog stools, wrack, and beach sand.* gelE* was the only virulence gene found among* E. faecium* from eelgrass.

**Figure 1:**
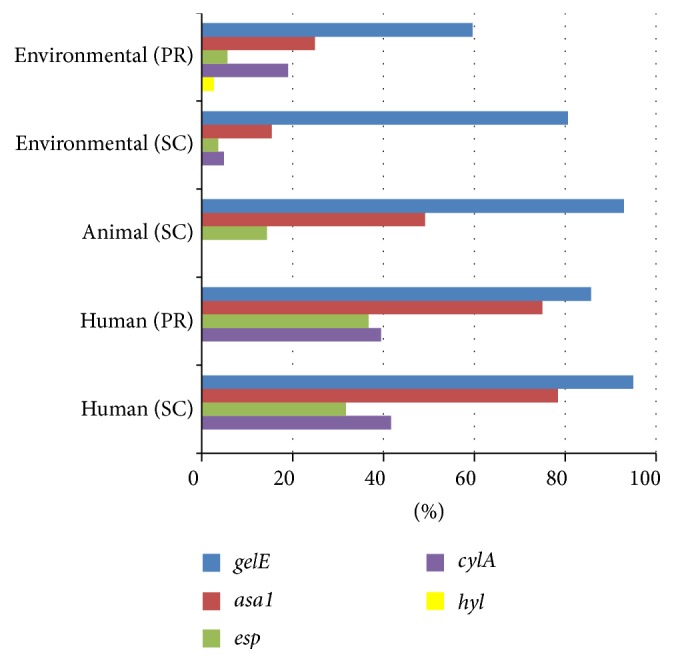
Distribution of virulence factor genes among* E. faecalis* isolates from environmental, animal, and human sources in Puerto Rico (PR) and Southern California (SC).

The distribution of enterococcal virulence genes was also compared based on categorizing the source of* E. faecalis* and* E. faecium* isolates as environmental, animal, and human.* E. faecalis* isolates from human specimens (clinical and nonclinical) from both geographic locations had a higher frequency of the five virulence genes overall (Figure 1).* gelE* was the most abundant virulence gene found among* E. faecalis* isolates from human, animal, and environmental sources (59.6% to 95%), followed by* asa1* (15.4% to 78.4%).* cylA* was found among 19.0% to 41.7% of* E*.* faecalis* human isolates and 4.9% to 19.0% environmental isolates and not detected in animal isolates.

**Figure 2:**
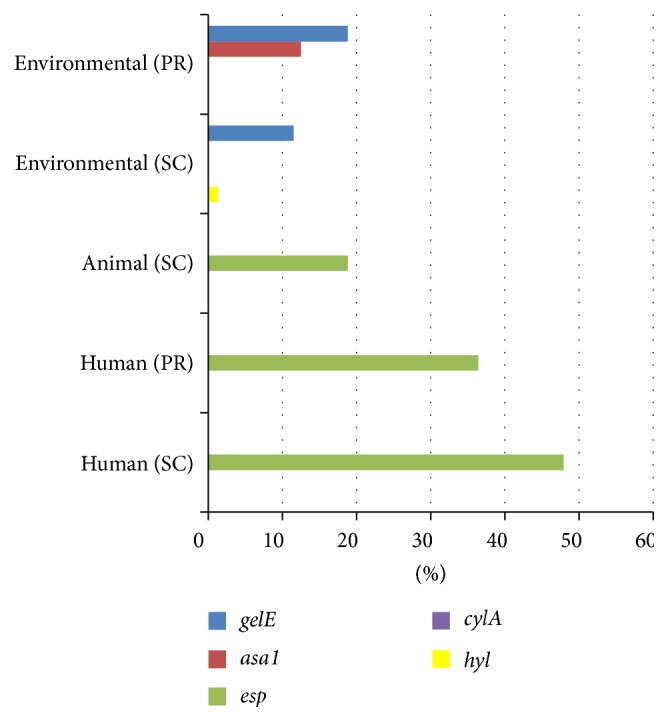
Distribution of virulence factor genes among* E. faecium* isolates from environmental, animal, and human sources in Puerto Rico (PR) and Southern California (SC).

*esp* was the most commonly found virulence gene detected among* E. faecium* isolates (0% to 47.9%), followed by* gelE* (0% to 18.8%),* asa1* (0% to 12.5%), and* hyl* (0% to 1.3%) (Figure 2). At both study sites, human derived* E. faecium* isolates had the highest frequency of* esp* (36.4% to 47.9%).

## 4. Discussion

*E. faecalis* and* E. faecium* obtained from multiple sources, including the beach environment, humans (clinical and nonclinical), animals, and birds in Southern California and Puerto Rico, harbored putative enterococcal virulence genes that differed in frequency depending on source. At both study locations, there was a higher prevalence of virulence genes among* E. faecalis* as compared to* E. faecium*. Among both species groups, virulence genes were less abundant among beach strains overall compared to human isolates, which was also consistent with a similar study conducted in Australia [35].

Enterococcal virulence genes* asa1* (aggregation substance) and* cylA* (cytolysin activator) were found among* E. faecalis* isolates from beach water, humans, dogs, and birds, indicative of strains with enhanced virulence potential.* asa1* and* cylA* were first identified in the genome of multidrug resistant* E. faecalis* strain MMH594 and have also been associated with* E. faecalis* pathogenicity islands [16, 36]. Aggregation substance is encoded on a sex pheromone plasmid and mediates aggregation between bacteria, enabling the transfer of plasmids [37]. Cytolysins are toxins secreted by bacteria that damage cell membranes, facilitating the infection process.* cylA* can be carried on a plasmid or occur on the bacterial chromosome [38].

The distribution of* asa1* and* cylA* among* E. faecalis* from human clinical specimens was 90% and 70%, respectively, of* E. faecalis* from SC as compared to 50% and 19%, respectively, of isolates from PR. These differences likely reflect variability in the types of clinical specimens analyzed from each study location; clinical isolates of* E. faecalis* from SC were obtained from rectal swabs, urine, blood, abscess, ascites, vagina, and joints; those from PR were obtained primarily from urine specimens.

*asa1* and* esp* were also found among* E. faecalis* strains in dogs and birds (mostly seagull), suggesting that they may be important reservoirs of strains that could potentially be transferred to humans.* esp* is thought to aid enterococci in evading the immune system and also form biofilm [36, 39], which facilities colonization of* E. faecalis* in acute urinary tract infections [14]. Animals and birds have been suggested as potential sources of virulent strains to humans;* gelE*,* asa1*,* esp*, and* cylA* were detected in fecal* E. faecalis* isolated from dogs at veterinary hospitals [40, 41], poultry [42], and ducks and wild geese [43]. The presence of these genes among* E. faecalis* strains from dogs and birds warrants further studies to assess potential human health risks.

Among the virulence genes analyzed,* gelE* was the most frequently detected and widely distributed among* E. faecalis* strains from multiple sources, including the environment which is consistent with previous studies [9, 44, 45].* gelE* is thought to enhance survivability of enterococci in extraintestinal environments [46].

In the beach environment,* E. faecium* was rare among enterococci identified from eelgrass, sewage influent, and dog samples, thus limiting the number of isolates that could be analyzed for virulence genes.* E. faecium* and* E. faecalis* were also rare or not detected in composite fecal samples from horses, goats, and pigs from PR, which is consistent with studies showing the low prevalence of these species in livestock [43, 47, 48]. Birds were rarely observed at the study beaches in PR, which precluded efforts to obtain enterococci isolates.

It is important to note that the presence of virulent strains among* E. faecalis* and* E. faecium* alone is not predictive of infection as there may be other mediators of pathogenicity that have yet to be elucidated [49]. It has been suggested that pathogenicity is also related to the ability of virulent strains to grow in high densities in the intestinal tract and spread to other sites in the body [50]. Host factors, such as predisposing medical conditions, immune status, and exposure to antibiotics, are also thought to play a role in the ability of enterococci to establish infection [51].

## 5. Conclusion

The low incidence of* asa1*,* esp*, and* cylA* among* E. faecalis* and* E. faecium* from the PR and SC beaches indicates that these virulence genes were not widely disseminated among strains found here, suggesting low potential health risks to humans. Still, the presence of* E. faecalis* and* E. faecium* harboring* asa1*,* esp*, and* cylA* suggests humans, birds, and dogs as potential sources of enterococci to beach water. Future surveys of enterococcal virulence genes at beaches should include those with different source inputs and populations of enterococci.

## Figures and Tables

**Table 1:** Sources of *E. faecalis* and *E. faecium* isolated from Southern California.

Source	Number of samples	Number of sites	Number of isolates		Total number of isolates
			*E. faecalis*	*E. faecium*	
Environmental					
Beach water	5	5	8	10	18
Urban runoff	10	5	20	8	28
Sand	5	5	0	5	5
Sea wrack	5	1	5	5	10
Eelgrass	7	1	5	3	8
Wastewater influent	4	4	6	3	9
Wastewater effluent	5	2	6	11	17
Human					
Human, healthy	18	NA	15	8	23
Human, clinical	10	Unk	10	5	15
Vancomycin resistant enterococci	Unk	Unk	0	10	10
Animal					
Dogs	7	7	7	3	10
Birds	16	2	9	8	17

Total	92		91	79	170

NA = not applicable.

Unk = unknown.

**Table 2:** Sources of *E. faecalis* and *E. faecium* isolated from Puerto Rico.

Source	Number of samples	Number of sites	Number of isolates		Total number of isolates
			*E. faecalis*	*E. faecium*	
Environmental					
Beach water	9	2	108	32	140
Septage	6	6	19	26	45
Human					
Human, healthy	9	1	5	4	9
Human, clinical	53	1	42	11	53

Total	77		174	73	247

**Table 3:** Distribution of virulence factor genes among* E. faecalis* isolates from Southern California (SC) and Puerto Rico (PR).

Source (number) of isolates	% (number) of isolates for the following virulence factor genes:											
	*gelE*		*asa1*		*esp*		*cylA*		*hyl*		None	
PR beach water (108)	98.1%	(106)	44.4%	(48)	11.1%	(12)	3.3%	(3)	0.0%	(0)	19.4%	(21)
SC beach water (8)	100.0%	(8)	12.5%	(1)	0.0%	(0)	12.5%	(1)	0.0%	(0)	0.0%	(0)
SC wrack (5)	100.0%	(5)	20.0%	(1)	0.0%	(0)	0.0%	(0)	0.0%	(0)	0.0%	(0)
SC eelgrass (5)	80.0%	(4)	0.0%	(0)	0.0%	(0)	0.0%	(0)	0.0%	(0)	20.0%	(1)
SC urban runoff (20)	70.0%	(14)	10.0%	(2)	5.0%	(1)	0.0%	(0)	0.0%	(0)	30.0%	(6)
SC sewage influent (6)	50.0%	(3)	50.0%	(3)	16.7%	(1)	16.7%	(1)	0.0%	(0)	33.3%	(2)
SC sewage effluent (6)	83.3%	(5)	0.0%	(0)	0.0%	(0)	0.0%	(0)	0.0%	(0)	16.7%	(1)
PR septage (19)	21.0%	(4)	5.3%	(1)	0.0%	(0)	5.3%	(1)	5.3%	(1)	73.7%	(14)
SC dog (7)	85.7%	(6)	42.9%	(3)	28.6%	(2)	0.0%	(0)	0.0%	(0)	0.0%	(0)
SC bird (9)	100.0%	(9)	55.5%	(5)	0.0%	(0)	0.0%	(0)	0.0%	(0)	0.0%	(0)
PR human, nonclinical (5)	100.0%	(5)	100.0%	(5)	40.0%	(2)	60.0%	(3)	0.0%	(0)	44.4%	(2)
SC human, nonclinical (15)	100.0%	(15)	66.7%	(10)	33.3%	(5)	13.3%	(2)	0.0%	(0)	0.0%	(0)
PR human, clinical (42)	71.4%	(30)	50.0%	(21)	33.3%	(14)	19.0%	(8)	0.0%	(0)	7.1%	(3)
SC human, clinical (10)	90.0%	(9)	90.0%	(9)	30.0%	(3)	70.0%	(7)	0.0%	(0)	10.0%	(1)

**Table 4:** Distribution of virulence factor genes among *E. faecium* isolates from Southern California (SC) and Puerto Rico (PR).

Source (number) of isolates	% (number) of isolates for the following virulence factor genes:											
	*gelE*		*asa1*		*esp*		*cylA*		*hyl*		None	
SC beach water (10)	0.0%	(0)	0.0%	(0)	0.0%	(0)	0.0%	(0)	0.0%	(0)	100%	(1)
PR beach water (32)	37.5%	(12)	25.0%	(8)	0.0%	(0)	0.0%	(0)	0.0%	(0)	62.5%	(20)
SC wrack (5)	0.0%	(0)	0.0%	(0)	0.0%	(0)	0.0%	(0)	0.0%	(0)	100%	(5)
SC eelgrass (3)	33.0%	(1)	0.0%	(0)	0.0%	(0)	0.0%	(0)	0.0%	(0)	67.0%	(2)
SC sand (5)	0.0%	(0)	0.0%	(0)	0.0%	(0)	0.0%	(0)	0.0%	(0)	100%	(5)
SC urban runoff (8)	50.0%	(4)	0.0%	(0)	0.0%	(0)	0.0%	(0)	0.0%	(0)	50.0%	(4)
SC sewage influent (3)	0.0%	(0)	0.0%	(0)	0.0%	(0)	0.0%	(0)	0.0%	(0)	100%	(3)
SC sewage effluent (11)	9.1%	(0)	0.0%	(0)	0.0%	(0)	0.0%	(0)	0.0%	(0)	90.9%	(10)
PR septage (26)	0.0%	(0)	0.0%	(0)	0.0%	(0)	0.0%	(0)	0.0%	(0)	100%	(26)
SC dog (3)	0.0%	(0)	0.0%	(0)	0.0%	(0)	0.0%	(0)	0.0%	(0)	100%	(3)
SC bird (8)	0.0%	(0)	0.0%	(0)	37.5%	(3)	0.0%	(0)	0.0%	(0)	62.5%	(5)
PR human, nonclinical (4)	0.0%	(0)	0.0%	(0)	0.0%	(0)	0.0%	(0)	0.0%	(0)	100%	(4)
SC human, nonclinical (8)	0.0%	(0)	0.0%	(0)	12.5%	(1)	0.0%	(0)	0.0%	(0)	87.5%	(7)
PR human, clinical (11)	0.0%	(0)	0.0%	(0)	72.7%	(8)	0.0%	(0)	0.0%	(0)	0.0%	(0)
SC human, clinical (5)	0.0%	(0)	0.0%	(0)	83.3%	(4)	0.0%	(0)	0.0%	(0)	16.7%	(1)
SC vancomycin resistant enterococci (10)	0.0%	(0)	0.0%	(0)	80.0%	(8)	0.0%	(0)	10.0%	(1)	10.0%	(1)
